# Coverage survey of typhoid conjugate vaccine among children aged 6 months to 15 years in an urban slum settlement of Lyari Town Karachi, Pakistan

**DOI:** 10.1371/journal.pone.0289582

**Published:** 2023-08-07

**Authors:** Rabab Batool, Sonia Qureshi, Zoya Haq, Mohammad Tahir Yousafzai, Rehana A. Salam, Rafey Ali, Tahira Sadaf, Miqdad Ali, Farah Naz Qamar

**Affiliations:** 1 Department of Pediatrics and Child Health, Aga Khan University Hospital, Karachi, Pakistan; 2 Center for Child, Adolescent, and Maternal Health, Faculty of Medicine and Health Technology, Tampere University, Tampere, Finland; 3 Liaqat National Hospital, Karachi, Pakistan; 4 Kirby Institute, University of New South Wales, Sydney, NSW, Australia; Health Services Academy, PAKISTAN

## Abstract

**Objective:**

To estimate the coverage rate of typhoid conjugate vaccine (TCV) among children aged 6 months to 15 years in Lyari Town Karachi, Pakistan.

**Methods:**

A cross-sectional survey was conducted to estimate the vaccine coverage of Typbar TCV in Lyari Town Karachi utilizing the World Health Organization (WHO) recommended rapid vaccine coverage assessment technique (30 clusters × 7 households). Sampling was powered at town level and multistage cluster sampling was used. Four union councils were randomly selected from a total of 11 and the survey was conducted in those union councils. After consent was obtained, parents of age-eligible children living in the selected union councils were invited to participate in the survey and information was collected on Typbar TCV vaccination status of children aged 6 months to 15 years.

**Results:**

Overall, 2325 children were included in the survey. The mean age of the participants was 7.60 ± 3.84 years. The ratio of males to females was equal in the survey sample; 1163 (50.02%) were male. In the total target population, 82% children were found to be vaccinated; however, the vaccination status could be verified for 80%. The vaccine coverage of TCV was comparable among the four union councils and the overall coverage of TCV vaccine in Lyari Town was found to be 80%. The coverage was significantly lower in younger children, 5% and 17% among children aged 6 months to < 2 years and 2 years to < 5 years respectively and 78% among children aged 5 years to 15 years.

**Conclusion:**

The overall immunization coverage rate with TCV was found to be satisfactory. Immunization coverage was comparable among both sexes and the selected union councils but it was relatively low among children in younger age groups.

## Introduction

Typhoid fever is a major contributor to the global enteric fever burden in low-and middle-income countries (LMICs) of Africa and Asia [1, 2]. This means that three-quarters of the world’s population is at risk of contracting it [3]. Typhoid accounts for 11 to 21 million cases of enteric fever, all of which are predominantly found in underprivileged regions [4]. The incidence of the disease is high in children, with at least one in five children having suffered from typhoid fever by the age of 10. In 2017, approximately 128,000 to 161,000 typhoid-related deaths occurred worldwide [4]. This variation can be explained primarily by differences in access to clean water and sanitation, population prevalence of chronic carriers, baseline immune status, and food hygiene [3]. Children are disproportionately affected by typhoid fever [5], with the highest incidence observed in school-going children and young adults in typhoid endemic settings such as Karachi, Pakistan where a rate as high as 1000 cases per 100,000 child years was reported in 2010 [6].

An outbreak of extensively drug resistant (XDR) typhoid fever was first reported in the Hyderabad district of Sindh Province, Pakistan in November 2016. It soon spread to neighboring cities and involved the entire province [6]. The circulating XDR strain of *Salmonella* Typhi haplotype was resistant to first and second-line antibiotics as well as third-generation cephalosporins [7]. From January 1, 2017 to February 16, 2020; 11,481 out of 16,869 (68%) cases were confirmed as XDR typhoid cases in Karachi by the Provincial Disease Surveillance and Response Unit (PDSRU). 69% percent of cases were reported in Karachi (the commercial capital of the country), 27% in Hyderabad, and 4% in other districts of the province [8]. In 2017, 63% of typhoid cases and 70% of typhoid deaths in Pakistan occurred in children younger than 15 years [7]. The high number of cases, coupled with limited treatment options, imposed significant clinical and economic repercussions, intensifying the urgency to expand primary preventive measures, such as vaccines, to curtail the detrimental effects of the disease and prevent the further spread of XDR typhoid [1, 9–11].

World Health Organization (WHO) prequalified Typbar TCV, to be the first typhoid conjugate vaccine (TCV). The prequalification of Typbar TCV has raised the hopes of controlling outbreaks by slowing the spread of XDR strains [5]. Furthermore, typhoid endemic countries are already licensed to use TCV and have been recommended by WHO to include the vaccine into the Expanded Program for Immunization (EPI) schedule for infants and children over six months of age [5]. Nevertheless, the comprehensive management of typhoid fever requires integration of typhoid vaccination with multipronged strategies for optimal disease control [12]. As a response to rapidly spreading typhoid outbreak in Karachi, AKU launched an outbreak response and initiated a mass immunization campaign in Lyari Town, where the highest number of XDR typhoid cases were reported. Between April 10, 2019 and October 24, 2019, a total of 87,993 children aged 6 months to 15 years were vaccinated with Typbar TCV vaccine in Lyari Town, Karachi.

Immunization coverage surveys aim to provide coverage estimates for a specific vaccine or a set of vaccines. In LMICs where the estimates of population denominator and the reported number of vaccinations are often inaccurate, coverage is an extensively used indicator of programme performance [13]. Surveys facilitate the assessment of coverage estimates by factors such as district, ethnicity, age, sex, paternal factors, socioeconomic class, or subnational region. Vaccination coverage surveys can assess the pockets of low coverage and regional disparities in immunization and provide data to plan future strategies and effective interventions to improve immunization coverage [14].

A vaccine coverage survey was conducted after the completion of vaccination campaign to estimate the coverage rate of TCV among age-eligible children, post-mass immunization campaign in Lyari Town Karachi, Pakistan before October 24, 2019.

## Materials and methods

To estimate the vaccine coverage of TCV, a cross-sectional survey was conducted between November 8, 2019 and November 18, 2019 in Lyari Town, Karachi. WHO-recommended rapid vaccine coverage assessment technique (30 clusters × 7 households) [15, 16] was used, and sampling was powered at town level. Multi-stage simple random sampling technique was applied. The first sampling stage consisted of random selection of a predetermined number of union councils (UCs), and 30 clusters were randomly selected per UC. Out of 11 UCs in Lyari, four were randomly selected for participation. Gridded high-resolution satellite maps were used to check the boundaries, potentially allowing UC borders to be overlaid on satellite images via Google Earth. The boundaries were verified using data provided by UC offices. Unfortunately, an up-to-date sampling frame for Lyari Town was not available. Different grid sizes were overlaid on satellite images of UCs maps for further segmentation of UCs into clusters. To estimate the appropriate size of each cluster and to make them comparable to enumeration areas (EAs), different sizes of clusters were verified by field team members (research associates, senior research assistants, field supervisors and data collectors) by visiting clusters and counting the households in 20 clusters. The range of households was reviewed until the desired number of households per cluster was reached. The mean number of households in each cluster finally equaled 200–250 units. All the clusters were exhaustive, non-overlapping and mutually exclusive. A 75 × 75m grid was generated using the fishnet tool in Aeronautical Reconnaissance Coverage Geographic Information System (ArcGIS). This grid was overlaid with UC boundaries to associate grid cells with specific UCs. [17]. Cluster identification numbers were assigned to each grid cell for unique identification and each cell of the grid was treated as a cluster if it met the criteria set by cluster definition. On the other hand, the whole town was split up into two categories; residential and non-residential (including commercial areas, industrial areas, recreational areas, and open spaces). This was performed using geospatial techniques along with in situ validation. The layer of residential zones was overlaid with grid cells with criteria of grid cells containing 75% and greater area with the residential layer designated as clusters. Each UC had an average of 60 clusters.

Finally, using a spatial sampling tool [18], seven random sample points were generated for the selection of households in each cluster.

In order to avoid compromising the representativeness of results and biasing estimates upward, field data collectors were left with no room to choose households for the survey of their own accord. Sketch maps were provided on electronic tablets and field team used these maps to navigate to the randomly selected households in each cluster. GIS locations of the households visited were also recorded at the time of survey. This method efficiently improved representativeness and facilitated supervision and external monitoring of adherence to the survey protocol by comparing the locations of households selected with those visited.

**Fig 1:**
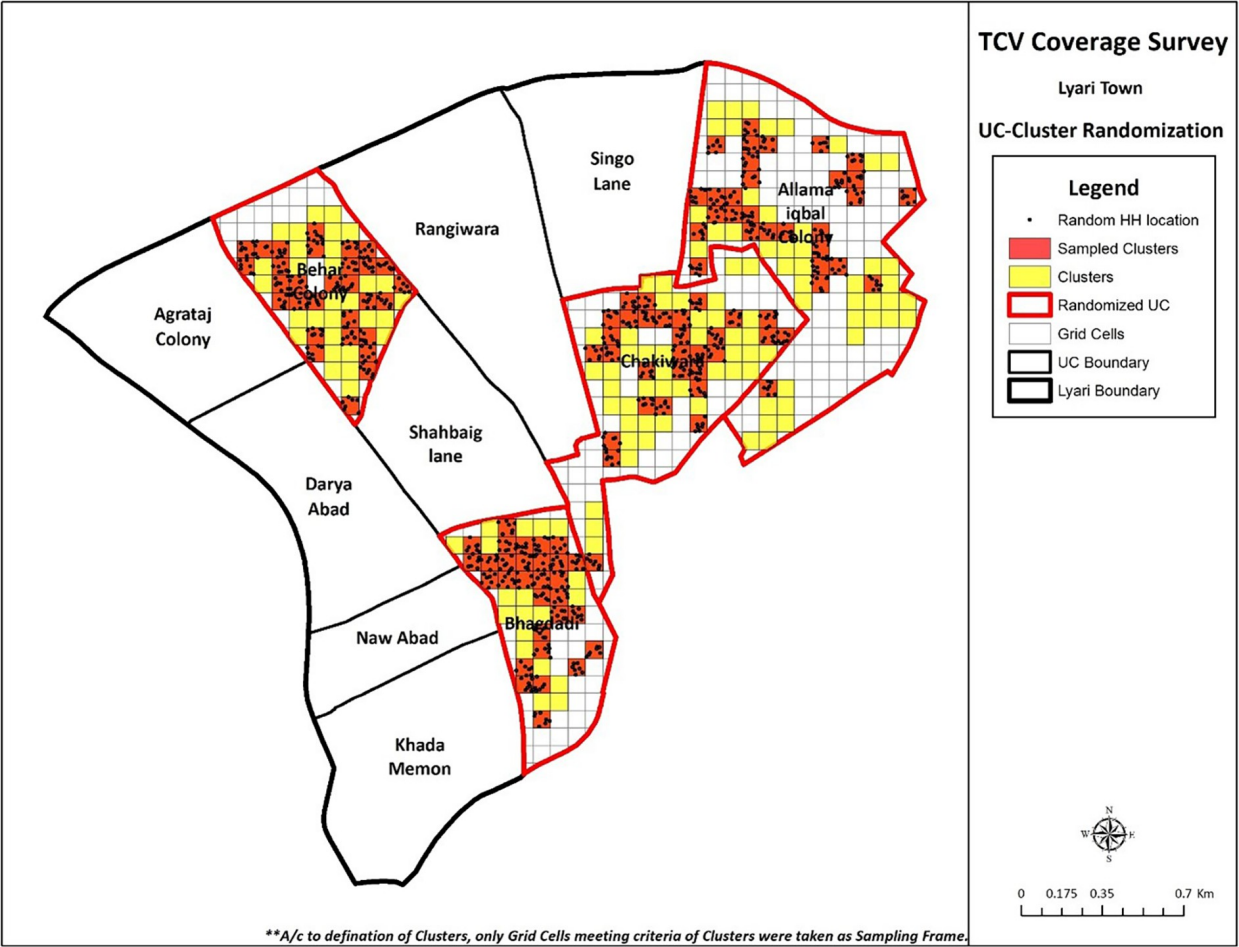
GIS map for coverage survey of TCV—Lyari Town, Karachi.

In each of the selected UC 210 households were included to obtain UC-level information on coverage rates with sufficient power for statistical calculations. A detailed GIS map for the coverage survey was created (Fig 1).

A trained team of research assistants accompanied by a local community member visited randomly selected points at each UC. If a household was selected but there was no age-eligible child in that household, or if the selected structure was a plot under construction or a non-residential building, the team skipped three households at a distance to approach the next closest household to check eligibility, repeating the process until an eligible household was found. Data were collected for every eligible individual who either slept in that household the previous night or lived there permanently. Parents of age-eligible children living in the selected union councils were invited to participate in the survey to obtain information on TCV vaccination status of children aged 6 months to 15 years old. Households were excluded if parents refused to provide consent, however, information such as the address, names, ages and genders of the occupants and respondents was maintained. All parents and primary caregivers/guardians (where applicable), in selected households were interviewed on behalf of their children. The team conducted and documented revisits to households where the respondents had been unavailable during the first visit. At least three visits were made to each household at different times on the same day. Neighbors were asked about their families’ whereabouts. When a home-based record was not available or was poorly filled (illegible or incomplete), vaccination documentation at the child’s usual healthcare facility(s) was consulted. This was performed in addition to asking for and recording the caretaker’s recollection of child’s vaccination history. The team asked about the place (school/health facility), date and time of vaccination, along with child’s details of school and class, date of birth, parent’s name, computerized national identity card and contact numbers. Records were retrieved from the vaccination database for cases with missed vaccination cards. The child did not need to be physically present at the time of the interview; however, a caretaker or knowledgeable guardian, ideally someone who could supply the child’s vaccination card, ought to be present to proceed with the survey. If no knowledgeable caretaker or vaccination card was available during the first visit, subsequent visits in the evening or any available time during the same day were scheduled before the interviewer left the cluster. Finally, the interview outcome for each visit was recorded.

### Assessment of vaccination status

Vaccination status was assessed using a two-fold method to ensure reliable results. First, the vaccination cards, where available, were used to determine the vaccination status of the child. Upon unavailability of vaccination card, the interviewer, to assess the child’s vaccination status, relied on caregiver’s memory of vaccine administration. The vaccination status was then confirmed using vaccination database. For unvaccinated children, caregiver’s information alone was used to record the vaccination status of the child.

### Data analysis for vaccine coverage survey

After extensive cleansing, data were imported into STATA version 16 for analysis. Descriptive analysis was performed to estimate the overall TCV-specific coverage, UC-level coverage rates and coverage among children of different age groups.

### Ethics statement

The study was approved by the Ethical Review Committee of Aga Khan University Hospital. (ethical identification number 0887). All participants voluntarily provided written informed consent to participate in the study. A child was enrolled in the study if a parent or legal guardian was willing and capable of providing informed consent. If the participant was seven years of age or older, child assent was also sought.

## Results

A total of 840 households were visited in Lyari Town Karachi. All households approached were included in the survey. Overall, 2325 children aged 6 months to 15 years were included in the survey, making the average number of children of desired age group per household to be 4 (standard deviation [SD] ± 2). The mean age of the participants was 7.60 [SD ± 3.84] years. The ratio of males to females was equal in the survey sample, 1163 (50.02%) were male.

Out of total, 1900 (82%) children were found to be vaccinated; however, the vaccination status could be verified for 1857 (79.87%) children either through vaccination cards or vaccination datasets. When asked about the vaccination cards, 978 (52.67%) parents/caregivers were able to show vaccination cards, while 446 (24.02%) parents/caregivers did not have vaccination cards, and 433 (23.32%) reported that despite having vaccination cards, they would be unable to produce them at the time of interview. If vaccination card was not available, vaccination status was verified from the vaccination data record, however, for 3% of the study sample, vaccination status could not be verified; TCV coverage percentage for verified and non-verified data were assessed separately. Upon inquiring about the preferred type of health facility for vaccination, 1954 (84.04%) of respondents mentioned that they preferred a public healthcare facility. We found that 29 (1.56%) children were administered the dose from Kharadar General Hospital, 118 (6.35%) were vaccinated at Lyari General Hospital, 146 (7.86%) were vaccinated at Janbai Aga Khan Secondary Care Hospital, 875 (47.12%) children were vaccinated at schools during the school vaccination campaign and 689 (37.10%) were vaccinated either at community camps or mobile vaccination camps.

A total of 1607 (69.12%) parents reported that the distance to reach the preferred vaccination center was < 2 km and 1228 (66.13%) of respondents mentioned that the time taken to reach the preferred vaccination center was < 15 minutes. 1421 (76.5%) of the surveyed parents reported taking their children by foot to a nearby health facility for immunization. Majority of parents (95.96%) were aware of the ongoing typhoid vaccination campaign in the area (Table 1).

**Table 1:** Characteristics of the children approached for participation in typhoid conjugate vaccine (TCV) coverage survey.

Participant’s characteristics	N (%)
**N**	2325
**Age (years)**	
Mean ± SD	7.60 ± 3.84
**Gender**	
Male	1163 (50.02%)
Female	1162 (49.98%)
**Vaccination status verified **	
Yes	1857 (79.87%)
No	468 (20.13%)
**Vaccination card available **	
Yes	978 (52.67%)
No	446 (24.02%)
Available but not shown	433 (23.32%)
**Parental preference regarding vaccination center**	
Public	1954 (84.04%)
Private	371 (15.96%)
**Health facility utilized for typhoid vaccination**	
Kharadar General Hospital	29 (1.56%)
Lyari General Hospital	118 (6.35%)
Janbai Aga Khan Secondary Care Hospital	146 (7.86%)
School	875 (47.12%)
Other (community camps/mobile vaccination campaign)	689 (37.10%)
**Mode of transportation to the preferred vaccination center**	
Car / bike	252 (13.57%)
Taxi / auto rickshaw	159 (8.56%)
Public transport	25 (1.35%)
By walk	1421 (76.52%)
**Distance from preferred vaccination center**	
< 2 km	1607 (69.12%)
2–4 km	434 (18.67%)
> 4 km	86 (3.70%)
Don’t know	198 (8.52%)
**Time to reach preferred vaccination center**	
< 15 minutes	1228 (66.13%)
15 minutes– 30 minutes	617 (33.23%)
> 30 minutes	12 (0.65%)
**Parental awareness regarding TCV campaign**	
Yes	2231 (95.96%)
No	94 (4.04%)

Vaccine coverage with a single dose of TCV was comparable between females and males; 80% (95% Confidence Interval (CI): 78%, 82%) versus 78% (95% CI: 77%, 82%).

The vaccine coverage of TCV was comparable among the four union councils: 77% (95% CI: 74%, 80%) in Baghdadi, 82% (95% CI: 79%, 85%) in Behar Colony, 87% (95% CI: 84%, 90%) in Chakiwara, and 74% (95% CI: 71%, 78%) in Allama Iqbal Colony. The overall coverage of Typbar TCV vaccine in Lyari Town Karachi was found to be 80% (95% CI: 78%, 81%) (Table 2).

**Table 2:** Summary of typhoid conjugate vaccine (TCV) coverage survey results among children 6 months to 15 years of age in Lyari Town, Karachi.

UC	Sampled Clusters	Sampled Households	Total Children	Children vaccinated	Children with verified vaccination status	Coverage Percentage, % (95% CI)	Coverage Percentage (verified vaccination status only), % (95% CI)
			6 months -15 years				
Baghdadi	30	210	637	515	490	81% (78%, 84%)	77% (74%, 80%)
Behar Colony	30	210	515	422	422	82% (79%, 85%)	82% (79%, 85%)
Chakiwara	30	210	545	481	472	88% (86%, 91%)	87% (84%, 90%)
Allama Iqbal Colony	30	210	628	482	466	77% (74%, 80%)	74% (71%, 78%)
**Grand Total**	**120**	**840**	**2325**	**1900**	**1850**	**82% (80%, 83%)**	**80% (78%, 81%)**

In addition, we assessed vaccination coverage among children of different age groups with verified vaccination status. Our findings revealed a significantly low coverage rate among younger children. TCV vaccination coverage rate was found to be 5% and 17% among children aged 6 months to less than 2 years and 2 years to less than 5 years respectively and 78% among children aged 5 years to 15 years (Table 3).

**Table 3:** Typhoid conjugate vaccine (TCV) coverage estimates among children of different age groups in selected union councils of Lyari Town, Karachi.

	Baghdadi	Behar Colony	Chakiwara	Allama Iqbal Colony	Total	p-value*
	N = 490	N = 422	N = 472	N = 466	N = 1850	
6 months—< 2 years	19 (4%)	15 (4%)	32 (7%)	34 (7%)	99 (5%)	0.009
2 - < 5 years	83 (17%)	78 (18%)	62 (13%)	91 (20%)	315 (17%)	
5–15 years	388 (79%)	329 (78%)	378 (80%)	341 (73%)	1,437 (78%)	

*Chi-square test p-value

## Discussion

The up-to-date vaccine coverage using a single dose of TCV was found to be 80% among children aged 6 months to 15 years in Lyari Town, Karachi. Only 52.67% parents were able to show the vaccination card at the time of interview, whereas vaccination status of the remainder of selected population was verified using vaccination database. Approximately 50% of vaccinated children received the dose via school-based vaccination campaigns.

Compared to routine immunization coverage in Sindh Province of Pakistan [19], the coverage of TCV was found to be higher in our sample. The 2017–18 Pakistan demographic and health survey (PDHS) revealed that 66% of children aged 12–23 months were administered all basic vaccines. TCV has recently been included into the EPI schedule and administered to children aged nine months along with the measles vaccine. With regard to measles vaccine coverage specifically, the estimated coverage of first measles containing vaccine (MCV) dose 1, administered at 9 months of age through the EPI schedule, was found to be 76% in 2017 PDHS. The coverage of second MCV dose 2—administered at 15 months through the EPI schedule was found to be 45% in 2017–2018 PDHS. The survey reported that 66.6% of children aged 24–35 months received two doses of measles vaccine. The overall vaccination coverage for all vaccines in Sindh was 49%. Considering these statistics, it can be hypothesized that even after official introduction of TCV into routine immunization schedule, the potential coverage of TCV may be low in Sindh. Between 2000 and 2018, approximately 232.5 million children received doses of MCV during supplementary immunization activities (SIAs); if TCV is included in SIAs, we can expect a higher coverage rate [19].

In our survey, we found that 52.7% of parents were unable to produce a vaccination card at the time of interview. However, 2017–2018 PDHS revealed that 85% of parents of children aged 12–13 months reported having a vaccination card; nonetheless, vaccination cards for only 63% of the vaccinated children could be seen at the time of interview [19]. This percentage is higher than our results from the vaccine coverage survey in Lyari Town, Karachi. This might be owed to the fact that Lyari is a slum settlement with high illiteracy rates and lack of proper awareness. This population is thus not very well representative of all populations in Pakistan, but rather specifically pertains to similar urban slum communities.

The coverage rate of TCV found in our survey is concordant with a similar survey of Vi PS (76%) post-mass immunization campaign, conducted in the towns of Sultanabad and Hijrat Colony, Karachi [20].

We found that 47.12% of children with a positive vaccination history for TCV were vaccinated via school-based vaccination campaigns. Studies have also indicated that school-based vaccination strategies are successful in increasing vaccine coverage [21–23]. Low-to-middle-income countries, where vaccination coverage is low, can greatly benefit from school vaccination programs to increase their uptake. Schools may offer advantages in reaching the adolescent population, who do not regularly attend health facilities. Implementing a focused immunization program in urban areas, utilizing schools as a platform, could potentially serve as a cost-effective strategy considering that typhoid incidence is high among school going children in Pakistan’s urban setting and leverages the observed sustained high immunization coverage rates achieved through previous experiences with measles catch-up campaigns conducted within school settings. This similarity was also observed in polio vaccination campaigns held within school settings. Such campaigns are especially beneficial in urban populations where school enrolment rates are much higher as compared to rural settings [24]. The reasons for the high prevalence of low class enrollments and school absenteeism in LMICs can be attributed to various barriers to education, such as food and housing insecurity, inadequate availability of instructors and academic resources, overcrowded classrooms, long distances to schools, poor infrastructure, and instances of violence [25, 26]. To ensure equitable access for the most vulnerable populations, school-based delivery of vaccines must be complemented by strategies to reach those not attending schools, such as mobile teams, outreach, and provision of vaccines at health facilities.

Moreover, the vaccine coverage of TCV was found to be low among children of younger age groups in Lyari Town Karachi. These findings align with those of TCV coverage survey conducted in Nepal, suggesting vaccine hesitancy among parents of younger children. However, further investigation is warranted to explore additional factors that may influence this trend, such as concerns regarding potential side effects of the parenteral vaccines and lack of trust in a newly introduced vaccine. A comprehensive understanding of reasons for low coverage in younger children will help to develop effective strategies and policies aimed at increasing the uptake of TCV [27].

In slum areas where vaccine hesitancy and refusal rates are high [27]; multi-dimensional strategies should be targeted, and community involvement should be maximized. House-to-house canvassing, community camps and mobile vaccination approach helped streamline our vaccination campaign by infiltrating the community and gaining trust which in turn increased vaccination coverage.

Through this survey, it was also observed that for targeting an age group beyond two years, a hospital-based vaccination campaign was insufficient in reaching the maximum target group since only 15.77% of children could be vaccinated in hospitals. Although we did not target only EPI facilities of the hospitals, staff was allocated to different sections of hospital waiting areas, pediatric outpatient departments and inpatient departments to counsel parents on need for vaccination; the vaccination uptake through hospitals remained low.

Outreach camps and mobile vaccination facilities in residential areas may be an effective strategy for increasing vaccine coverage rates in younger children. The cost for transportation and distance to service delivery points are the contributing factors to low vaccine coverage in resource-scarce settings [28]. These issues were well addressed through mobile community camps during mass immunization campaign in Lyari Town Karachi [29]. Majority of parents/caregivers reported that the distance from vaccination center was less than 2 km and that they reached the vaccination center by walking which took less than 15 minutes. In TCV mass immunization campaign, 84.22% of children were vaccinated outside the health facilities.

Another study assessed the coverage, safety, and logistic feasibility of a mass immunization campaign outside the local immunization program setting among pediatric population of urban squatter settlements in Karachi [20]. The results of the mass immunization campaign in two residential areas of Karachi showed that a large-scale vaccination program had good acceptance (74% vaccine coverage) and posed no major safety problems. Thus, a mass vaccination campaign in residential areas is logistically appropriate and safe [20].

In light of technological advancements, practices such as “spinning the pen” or “bottles” employed to gauge coverage surveys should be considered obsolete. These methods are complicated, time-consuming and may produce biased estimates [16]. We utilized a two-stage sampling technique and a modified GPS method, which is superior and provides unbiased estimates of vaccine coverage. The method we implemented in our coverage survey is a modified version of conventional EPI coverage survey methods following WHO 30 × 7 cluster survey design, focusing on reducing the potential for selection bias due to fieldworker practices and improving the accuracy and precision of survey results. In general, we used probability sampling methods and in the absence of quality census data and lists of enumeration areas for the sampling frame, this method served as a rational use of resources to conduct survey in a resource-scarce setting. The survey was planned well in advance to allow time to create sketch maps and develop softwares for electronic devices. A multidisciplinary team or steering committee comprising GIS and statistical experts was formed to oversee the survey, geographic information systems (GIS), and maps, provide guidance to field team throughout the field operations, and identify any potential gaps or issues requiring attention. The steering committee updated the coverage dashboard everyday, and it was reviewed by field supervisors on a daily basis.

Introduction of new vaccines in developing countries, particularly to the most impoverished, requires basic research, clinical evaluations, epidemiological assessments, policy and economic research, establishment of production facilities, sound regulatory systems, procurement mechanisms, and distribution capabilities. In areas where typhoid fever is a serious and widespread problem, TCV vaccination appears to be the most promising strategy for controlling typhoid fever. The mass vaccination campaign described here revealed that conducting a mass immunization outside of the EPI program and infrastructure is feasible and acceptable and could potentially be implemented in the institutionalization of TCV vaccination in public healthcare system.

## Conclusion

The overall immunization coverage rate with TCV was satisfactory. Immunization coverage within the surveyed union councils was comparable but significantly lower among children of younger age groups. There was no difference in the immunization status of both sexes. The results of coverage survey are encouraging and suggest that mass immunization campaigns outside the EPI program and infrastructure can be successfully implemented with overall good acceptance rates in Pakistan.

Up-to-date record keeping throughout the duration of vaccination campaigns coupled with coverage surveys can provide an accurate estimation of vaccination coverage. The impact of mass vaccination should be pre-planned based on the updated incidence rates and estimated target population. Data from vaccination coverage surveys can help identify pockets of low coverage and assess the indirect protection, “herd immunity”. The total vaccine effectiveness combines direct and indirect effects. However, it is uncertain whether TCV vaccines induce indirect protection. In future, it will be important to thoroughly explore the combined direct and indirect effects of TCV.

## Supporting information

S1 Questionnaire (DOCX)
