# Supplementary material for: Coverage survey of typhoid conjugate vaccine among children aged 6 months to 15 years in an urban slum settlement of Lyari Town Karachi, Pakistan
Source: PLoS One. 2023 Aug 7;18(8):e0289582. doi: 10.1371/journal.pone.0289582 (PMC10406246; doi:10.1371/journal.pone.0289582)
Supplement: S1 Questionnaire — (DOCX) [file pone.0289582.s001.docx]

Department of Paediatrics and Child Health

Aga Khan University

========================================================================

**TCV Impact Assessment study: Form - VACCINE COVERAGE SURVEY**

**Study ID:**

**Household Identification:**

**Town:** 01 Lyari **Union Council**: **Para/Mohalla/Neighborhood**

02 Saddar use code list of Union councils provided Use codelist of Para provided

GIS Coordinate:

Longitude:

Latitude:

*Town Union Council Para/Mohalla/neighborhood*

========================================================================

SECTION A: GENERAL INFORMATION

1. Is there any child or children born between _____ and _______, in this house? Yes 🞏 No 🞏

(If yes, please continue. If no, please complete respondent’s name, address, interviewer name, date of interview, and write ineligible household in comments box)

- 1. If yes, how many:

1. Has the verbal consent taken from parent / head of household for this interview: Yes 🞏 No 🞏
   1. If no, then specify the reason: __________________________________
2. Name of respondent________________________________________________

*(The respondent should be parent / primary caretaker of the child)*

1. Address__________________________________________________________
2. Are you aware of the ongoing Typhoid vaccination campaign in your area? Yes 🞏 No 🞏
3. Has your child ever been vaccinated against typhoid disease? Yes 🞏 No 🞏

6a. If yes by whom? 🞏 Vaccination center (EPI) 🞏 KGH Hospital 🞏Other (specify)_______________

1. What is the approx. distance of this household from the above mentioned vaccination place? (*Please check one*)

< 2 Kilometers 2 to 4 Kilometers >4 Kilometers 99. Don’t know

1. Which facility is utilized by your family for vaccination of children? (*Check any one that is mostly utilized)*

Govt. health center Private clinic /hospital KGH hospital Other specify: _______

1. Date of TCV vaccination:

2

0

1. Enrollment ID as per TCV vaccination card:
2. Date of interview:

2

0

Day Month Year

1. Status of interview: (Check any one) Completed Not completed

**Comments by Interviewer:**

|  |
| --- |

Name of interviewer ______________________________ Interviewer’s code:

2

0

Signature of Field Supervisor and date: ________________

Day Month Year

SECTION B: REASONS FOR NOT Vaccinating

(Ask the primary care taker, about the reason for not vaccinating for each child, if answer to Question 5 (TCV Vaccination) is “NO”)

| **Ask only one question:**  **“Why was the child [name(s) of the child] not vaccinated for typhoid disease?”**  **Mark (X) in the response columns** | | | | | |
| --- | --- | --- | --- | --- | --- |
| **S.#** | **Categories** | **Coding** | **Order of children** | | |
| 1 | Child number | | 1 | 2 | 3 |
| 2 | Sex (Male / Female) | |  |  |  |
| 3 | TCV Immunization status (copy from Q # 5 section A) | Immunized |  |  |  |
|  |  | Not Immunized |  |  |  |
| 4 | Lack of information | 1. Unaware of the need for immunization |  |  |  |
|  |  | 1. Fear of side reactions |  |  |  |
|  |  | 1. Other specify: |  |  |  |
|  | Lack of motivation | 1. No faith in immunization |  |  |  |
|  |  | 1. Rumors |  |  |  |
|  |  | 1. Other specify: |  |  |  |
|  | Obstacles | 1. Place of immunization too far |  |  |  |
|  |  | 1. Vaccinator absent/ did not come |  |  |  |
|  |  | 1. Vaccine not available |  |  |  |
|  |  | 1. Mother too busy |  |  |  |
|  |  | 1. Child ill – not brought |  |  |  |
|  |  | 1. Other specify: |  |  |  |

**Section C: Parent awareness and attitudes towards Typhoid vaccination (Tick appropriate box)**

SECTION C1: Personal Typhoid diseases experience

| **ID** | **Statement** | | **Yes** | **No** |
| --- | --- | --- | --- | --- |
|  | Have you personally seen someone with Typhoid? | |  |  |
|  | Do you know of someone in your family or community who had Typhoid? | |  |  |
|  | Have you ever delayed having child get a vaccination for reasons other than illness or allergy? (skip the question if child is not vaccinated ) | |  |  |
| 3.a | If yes, then why? | | List all reasons in order of priority | |
|  | Have you ever decided not to have your child get a vaccination? | |  |  |
| 4.a | If yes, then why? | | List all reasons in order of priority | |
|  | Would you want your child to get typhoid vaccination? | |  |  |
|  | Do you know the location of vaccination services? | |  |  |
|  | Do you know the time when vaccination services are offered? | |  |  |
|  | What do you think what are the side effects of vaccination among children? | | | |
| 12.a | If yes, what are these? (Mark as appropriate). | 1. Fever 2. Pain at site of injection 3. Pustule 4. Irritability 5. Disturbed sleep 6. Inappropriate feeding behavior 7. Any other (specify) | | |

SECTION C2: VPD RISK, SAFETY AND VACCINE EFFICACY PERCEPTIONS

Directions: For the following statements, please select one of the following: “Strongly Agree”, “Agree”, “Not Sure”, “Disagree” or “Strongly Disagree”

| **ID** | **Statement** | **Strongly agree** | **Agree** | **Not Sure** | **Disagree** | **Strongly disagree** |
| --- | --- | --- | --- | --- | --- | --- |
|  | Children get more vaccinations than are good for them |  |  |  |  |  |
|  | Healthy children do not need vaccinations. |  |  |  |  |  |
|  | vaccinations do more harm than good |  |  |  |  |  |
|  | It is better for my child to develop immunity by getting sick than to get a vaccination |  |  |  |  |  |
|  | I should be allowed to selectively choose the vaccines which I believe my child needs |  |  |  |  |  |
|  | It is better for my child to receive two injectable vaccinations in 1 visit rather than 1 injectable vaccination in 2 visits |  |  |  |  |  |
|  | I believe many of the illness which vaccinations prevent are severe |  |  |  |  |  |
|  | When I see a child or a picture of a child with either Polio, Diphtheria, Pertussis (Whooping cough), Tetanus, Hepatitis B, Pneumonia, Meningitis, or Measles, I am reminded of the need for vaccination |  |  |  |  |  |
|  | When my child is vaccinated, it benefits my entire community by reducing the spread of disease |  |  |  |  |  |
|  | When a parent refuses to vaccinate a child, it harms the entire community through risk of disease |  |  |  |  |  |
|  | I am concerned my child might have a serious side effect from a vaccination |  |  |  |  |  |
|  | Following the vaccination schedule is a good idea for my child |  |  |  |  |  |
|  | Parents who do not vaccinate their child should be penalized with a monetary fine |  |  |  |  |  |
|  | I would like to be a volunteer advocate for vaccination in my community if I am trained |  |  |  |  |  |

SECTION C3: INFLUENCES ON PARENTS’ DECISION ABOUT VACCINES

| **ID** | **Question** سوال | **Options** انتخابات | |
| --- | --- | --- | --- |
|  | Select your top three sources of information you trusted when you decided whether to vaccinate your child ***( Check up to three options)*** |  Mother-in-law   Husband   Other family member(s)   Radio/media   Healthcare provider   School teacher   Information video shown |  Television   Posters at local vendors   Friends   Religious leaders and organizations   SMS   Any other, specify |
|  | What times do you generally listen to radio? *(check all that apply)* |  Early morning (before 9am)   Mid-morning (9am-12pm)   Early afternoon (12pm-2pm) |  Afternoon (2pm-6pm)   Evening (after 6pm)   Never / I do not listen to the radio |
|  | What is your preferred language when listening to the radio? *(check only one)* |  Urdu   Punjabi   Pashto   Sindhi |  Balochi   English   Other____________ |
|  | What is your preferred language when viewing posters with healthcare messages? *(check only one)* |  Urdu   Punjabi   Pashto   Sindhi |  Balochi   English   Other, ___________ |

Directions: For the following statements, please select one of the following: “Strongly Agree”, “Agree”, “Not Sure”, “Disagree” or “Strongly Disagree”

| **ID** | **Statement** بيان | **Strongly agree** | **Agree** | **Not Sure** | **Disagree** | **Strongly disagree** |
| --- | --- | --- | --- | --- | --- | --- |
|  | I am able to openly discuss my concerns about vaccinations with my child’s healthcare provider |  |  |  |  |  |
|  | If my healthcare provider tells me vaccines prevent disease, I am more likely to have my child vaccinated |  |  |  |  |  |
|  | I am not well-treated by healthcare providers when I take my child for vaccination services |  |  |  |  |  |
|  | Private health facilities provide higher quality vaccination services than public health facilities |  |  |  |  |  |
